# Human placenta-based genome-wide mRNA sequencing to identify TEK/IGF1/CSF1/ANGPT2 as crucial segments in the pathogenesis of pre-eclampsia

**DOI:** 10.3389/fgene.2022.944932

**Published:** 2022-09-08

**Authors:** Lifeng Wang, Lin Zhang, Yuqin Fan, Yanjie Peng, Dandan Song, Jinfeng Fu, Xietong Wang

**Affiliations:** ^1^ Obstetrical Department, Shandong Provincial Hospital, Shandong University, Jinan, China; ^2^ Obstetrical Department, Key Laboratory of Birth Regulation and Control Technology of National Health Commission of China, Maternal and Child Health Care Hospital of Shandong Province, Jinan, China; ^3^ Clinical Medical Research Center for Women and Children Diseases, Maternal and Child Health Care Hospital of Shandong Province, Jinan, China

**Keywords:** pre-eclampsia, high-throughput sequencing, pathogenesis, etiology, diagnosis

## Abstract

Pre-eclampsia is a pregnancy-specific disease commonly occurring in late pregnancy and has always been threatening maternal and fetal lives, yet the etiology and pathogenesis of pre-eclampsia are still uncertain. To depict the overall changes of genes at the genome-wide level and identify potential biomarkers for early diagnosis of pre-eclampsia, we conducted this study by collecting placenta samples donated by six pregnancy women, among whom three healthy women were included as controls and three women were diagnosed with pre-eclampsia. The placental sample tissues were then subjected to high-throughput sequencing. Furthermore, we proceeded with bioinformatics analysis and formulated the hypothesis of pre-eclampsia development and verified the potential targets of pre-eclampsia by immunohistochemistry. Demographically, we found that the baseline characteristics of study subjects were highly homogeneous except for gestational weeks and blood pressure, where the blood pressure was higher and gestational weeks were shorter in the pre-eclampsia group (systolic blood pressure 123.33 ± 4.62 vs. 148.67 ± 3.79 mmHg, *p* = 0.046; diastolic blood pressure 79.00 ± 5.20 vs. 88.33 ± 2.89 mmHg, *p* = 0.068; gestational weeks 39.33 ± 1.03 vs. 35.76 ± 2.41, *p* = 0.050). Specific pathological changes were identified, shown as syncytial knots, fibrinoid necrosis, perivillous fibrin deposition, and vasculitis. For high-throughput sequencing, a total of 1,891 dysregulated genes were determined, of which 960 genes were downregulated and 931 genes were upregulated. The bioinformatics analysis indicated that these genes, with different molecular functions in different parts of cells, were primarily responsible for endothelium development and vascular process in the circulatory system, and more than 10 signaling pathways were involved. By focusing on the PI3K-Akt signaling pathway, Rap1 signaling pathway, and disease enrichment analysis item pre-eclampsia, TEK, CSF1, IGF1, and ANGPT2 were identified to promote the development of pre-eclampsia. After confirming the placental expression of these genes at the protein level, we proposed the pathogenesis of pre-eclampsia as follows: the downregulation of TEK, CSF1, IGF1, and ANGPT2 may inhibit trophoblast proliferation and affect the remodeling of spiral arteries, causing maternal and fetal malperfusion and impeding nutrient exchange, thereby leading to clinical manifestations of pre-eclampsia.

## Introduction

Pre-eclampsia is a pregnancy-specific physical disorder commonly characterized by the new-onset increased hypertension, proteinuria, and other end-organ damage after 20 weeks of gestation (Phipps et al., 2019). According to data released by the World Health Organization (WHO), pre-eclampsia occurs in an estimated 4.6% of all pregnancies, ranging from 2% to 15% worldwide, and is causally associated with maternal and fetal morbidity and mortality (Villar et al., 2009). In particular, pre-eclampsia has been previously reported to increase perinatal mortality by five-fold, and it is also the risk factor for 15% of preterm birth primarily due to an indicated delivery (Fox et al., 2019). Often, pre-eclampsia leads to persistent dysregulation of systemic physiology such as short-term complications of prematurity of the fetus, fetal growth restriction (FGR), low birth weight, and perinatal death (Crispi et al., 2018; Groten et al., 2019). Meanwhile, long-term complications of pre-eclampsia are commonly defined in the adult as cardiovascular diseases, neurodevelopmental disorders, and metabolic syndromes such as obesity, hypertension, and diabetes mellitus (Pittara et al., 2021; Barrea et al., 2022). Currently, there are no effective treatments for pre-eclampsia except timely terminating pregnancy, and the removal of the placenta seems to be the resolution of clinical manifestations of pre-eclampsia in most conditions, but pre-eclampsia complications may persist for a limited period postpartum.

In spite of severe threats caused by pre-eclampsia to public health and economic burden, the etiology and pathogenesis of pre-eclampsia are largely unknown. Over the past half-century, numerous studies from different basic, clinical, and epidemiologic aspects have been conducted to reveal potential risk factors leading to pre-eclampsia, and people now have come into consensus that pre-eclampsia is most likely to be a common final syndrome resulting from heterogeneous causes (Marinello et al., 2020; McCarthy et al., 2021; Miller et al., 2022). To date, the known risk factors of pre-eclampsia are extremes of age, obesity, smoking, drinking, history or family history of pre-eclampsia, diabetes mellitus, hypertension, poor socioeconomic status, multiple pregnancies, parity, and other factors (Machano and Joho, 2020). For example, accompanied by an adipose metabolic disorder, there is compelling evidence indicating that obesity is tightly associated with increased inflammation, insulin resistance, and oxidative stress during pregnancy, and suffering from obesity would increase the risk of pre-eclampsia by three-fold (Roberts et al., 2011). However, none of these risk factors exhibits adequate sensitivity and specificity to predict pre-eclampsia at the early stage of pregnancy.

Basic studies concerning the pathogenesis of pre-eclampsia highlight emerging insights into its diagnostic biomarkers, based on which several hypotheses have been raised to indicate the formulation of pre-eclampsia, and the most popular ones are immune disorder, vascular endothelium injury, and oxidative stress (Mannaerts et al., 2018; Afkham et al., 2019; Lu and Hu, 2019; Chappell et al., 2021; Wei et al., 2021). Consistently, these pathogenetic hypotheses share the same theoretical basis that pre-eclampsia can disrupt the biological function of the placenta, thereby inhibiting maternal–fetal exchange of nutrients and oxygen. In addition, the physical immune disorder causes inadequate remodeling of spiral arteries by extravillous trophoblasts, resulting in the manifestation of pregnancy complications such as maternal and fetal vascular malperfusion and leading to fetal hypoxia and damage to proteins, lipids, and DNA (James-Allan et al., 2018; Staff et al., 2022). Moreover, a recent review shows that placental oxidative stress is associated with poor placental perfusion and induces the release of serum-soluble vascular endothelial growth factor (VEGF). Also, oxidative stress can enhance the expression of soluble fms-like tyrosine kinase-1 (sFlt-1), soluble endoglin (sEng), and pro-inflammatory cytokines like interleukin- 1 (IL-1), IL-6, IL-12, and IL-18, but it decreases the expression of anti-inflammatory cytokines such as IL-10 (Guan et al., 2022). Although the alteration of these emerging molecules can be greatly attributed to the development of pre-eclampsia, the pathogenesis of pre-eclampsia can be hardly elucidated by one or several of them, and a knowledge gap between current findings, overall perspective of pre-eclampsia development, and early diagnosis of pre-eclampsia still exists.

To grasp the overall alterations of genes at the genome-wide level and identify potential biomarkers for early diagnosis of pre-eclampsia, we profiled the expression of placental mRNAs using high-throughput sequencing technology and identified the differentially expressed genes. Moreover, we adopted functional and disease enrichment analyses and proposed the assumption of pre-eclampsia pathogenesis. The expression of genes that were assumed to play dominant roles was further detected using immunohistochemistry assay. Data obtained in this study would contribute to a better understanding of the etiology and pathogenesis of pre-eclampsia as well as its early diagnosis and clinical treatment.

## Materials and methods

### Study subjects

Three women with pre-eclampsia and three women with normal single pregnancy who visited Maternal and Child Health Care Hospital, China, were enrolled in this study. A pregnant woman was diagnosed with pre-eclampsia when the presence of a new-onset of hypertension and proteinuria after 20 weeks of gestational age in accordance with the American College of Obstetricians and Gynecologists. In addition, pre-eclampsia can also be defined when the patients are in the absence of proteinuria but with the new-onset hypertension accompanied by the new onset of any of the following: thrombocytopenia, renal insufficiency, impaired liver function, pulmonary edema, new-onset headache unresponsive to medication and not accounted for by alternative diagnoses, or visual symptoms (Espinoza et al., 2020). Hypertension was defined as systolic blood pressure (SBP) > 140 mmHg or diastolic blood pressure (DBP) > 90 mmHg on two occasions at least 4 h apart. Also, an SBP >160 mmHg or DBP >110 mmHg was also considered to satisfy the requirement for blood pressure when diagnosing pre-eclampsia. Proteinuria was determined as protein ≥300 mg in urine collected over 24 h; gestational age was calculated based on the date of the last menstrual period and confirmed with early ultrasound examination. Information on risk factors associated with pre-eclampsia was recorded, including maternal age, pre-pregnancy BMI, pre-delivery BMI, FGR, gravidity, parity, abortion, historical live births, delivery arms, combination of anti-hypertensive drugs, neonatal birth weight, sex of the newborn, and baby Apgar scores. Exclusion criteria were set as follows: multiple gestations, drug abuse, and complication of chronic hypertension, diabetes, renal, acute intrauterine infection, autoimmune, and hepatic diseases. The study was approved by the Ethics Committee of Maternal and Child Health Care Hospital of Shandong Province (No. 2021-112), and all subjects provided informed consent.

### Placenta sample collection and preparation

The placenta tissues of the maternal surface were collected at 1.0 × 1.0 × 1.0 cm size immediately after delivery, with three samples in parallel for each subject. The tissues were washed with normal saline and placed in liquid nitrogen, 4% (*v*/*v*) paraformaldehyde, and TRIzol for downstream experiments, respectively.

### High-throughput sequencing

A total of six placenta samples were subjected to high-throughput sequencing on an Illumina HiSeq X Ten (Illumina), divided into the normal control and pre-eclampsia group with three each, and the high-throughput sequencing was adopted in a two-step manner with the help of Genergy Bio-Technology (Shanghai, China): RNA extraction and quality control and RNA library preparation and sequencing. In brief, the total RNA was first extracted, and RNA degradation and contamination were checked. Then, RNA integrity was examined via the RNA 6000 Nano Kit on an Agilent 2100 Bioanalyzer, and a strand-specific library for ribosomal RNA removal was constructed based on the NEBNext^®^ Ultra™ II Directional RNA Library Prep Kit for Illumina^®^ (NEB) with the Ribo-Zero rRNA Removal Kit Reference (Illumina, Madison, MI, United States).

### Bioinformatics analysis

The sequencing results were obtained in the format of count of the genes, and gene counts were first normalized and then log2 trans-formatted. The homogeneity of the samples was checked and visualized at the quality control stage using box plot, principal component analysis, and clustering analysis. The differences in genes between groups were determined via the limma test, and the differentially expressed genes were identified by the commonly used pre-set conditions: an absolute value of the log2 (fold change) ≥ 1 and *p* value <0.05. Utilizing the “clusterProfiler” package embedded in R statistical software, we conducted the bioinformatics analysis from four aspects, namely, Gene Ontology (GO), Kyoto Encylopaedia of Genes and Genomes (KEGG), gene set enrichment analysis (GSEA), and disease enrichment analysis (DES).

### Hematoxylin & eosin staining

After fixation with 4% paraformaldehyde over 48 h, the placenta tissues were decalcified and embedded in paraffin and then sliced into serial sections of 4 μm thickness. Subsequently, the tissue sections were deparaffinized in xylene and washed with gradient ethanol. An adequate volume of hematoxylin was added to stain the nuclei in blue for 10 min and then eosin to stain the cytoplasm in red for 1 min. After processing through dehydration and transparent and neutral gum sealing, the placenta tissues were observed under an optical microscope (Olympus, Tokyo, Japan), and the representative fields were captured and exhibited.

### Immunohistochemistry assay

The placenta tissue sections were prepared in line with protocols described in HE staining and then processed with heat-induced antigen retrieval. Subsequently, the tissues were incubated with specific primary antibodies of angiopoietin 2 (ANGPT2), colony-stimulating factor 1 (CSF1), insulin-like growth factor 1 (IGF1), and TEK at 1/500 dilution (Proteintech, Wuhan, China) and 4°C overnight. After removing the primary antibody, the tissues were incubated with the goat-anti-rabbit secondary antibody at 1/25,000 dilution (Zhongshan Golden Bridge, Beijing, China). DAB color developing solution was prepared in advance and dropped onto the tissues, and the color developing time was controlled under the microscope. The staining process was terminated under the tap water when the positive area was stained in brownish yellow. The sections were then counterstained with hematoxylin, differentiated with hematoxylin differentiation solution, and mounted with neutral resin size.

### Statistical analysis

All data obtained in this study were compared and visualized using R version 4.1.0. The continuous variables were represented as mean ± standard deviation, and differences between groups were compared using Student’s *t*-test. For comparison of frequency variables, a chi-squared test or Fisher exact test was applied. A *p*-value <0.05 was considered statistically significant.

## Results

### Demographic characteristics of study subjects

A total of six pregnant women were enrolled in this study, each three for the control group and the pre-eclampsia group. Demographically, the age of women in the control group was 27.67 ± 4.51 years, ranging from 25 to 30 years, lower than that of the pre-eclampsia group (32.33 ± 3.06 on average, ranging from 31 to 34 years). The pre-pregnancy BMI values for the two groups were 19.80 ± 3.41 vs. 24.77 ± 3.26, the gestational weeks were 39.33 ± 1.03 vs. 35.76 ± 2.41, the SBP values were 123.33 ± 4.62 vs.148.67 ± 3.79 mm Hg, and the DBP values were 79.00 ± 5.20 vs. 88.33 ± 2.89 mm Hg. None of the control was diagnosed with FGR, while two of three subjects were diagnosed with FGR in the pre-eclampsia group. For historical information on previous pregnancy, the subjects of both groups were found to be with 1–3 frequencies of gravidity, parity, abortion, and live births. The cesarean delivery arm was applied to all subjects of the pre-eclampsia group, and one of three subjects in the control group used this delivery pattern. For details of the fetuses, the average neonatal birth weight of the two groups was 3,140.00 ± 390.00 vs. 2353.33 ± 859.90 g, and the number of female babies was 1/3 vs. 2/3 in the two groups. The details are shown in Table 1.

**TABLE 1:** Baseline characteristics of study subjects.

	Control (*N* = 3)	Pre-eclampsia (*N* = 3)	*p*-value
Age (years)	28.00 [25.50; 30.00]	33.00 [31.00; 34.00]	0.127
Pre-pregnancy BMI (kg/m^2^)	20.80 [18.40; 21.70]	23.80 [22.95; 26.10]	0.127
Pre-delivery BMI (kg/m^2^)	29.20 [25.60; 29.85]	28.70 [28.20; 29.95]	0.827
Gestational age at delivery (weeks)	39.86 [39.00; 39.93]	36.86 [34.93; 37.14]	0.050
Fetal growth restriction			0.400
No	3 (100.00%)	1 (33.33%)	
Yes	0 (0.00%)	2 (66.67%)	
Gravidity	3.00 [2.50; 3.00]	3.00 [2.00; 4.00]	0.817
Parity	1.00 [1.00; 1.50]	2.00 [1.50; 2.50]	0.346
Abortion	1.00 [1.00; 1.50]	1.00 [0.50; 1.50]	0.637
Live births	1.00 [1.00; 1.50]	2.00 [1.50; 2.50]	0.346
Systolic blood pressure (mmHg)	126.00 [122.00; 126.00]	147.00 [146.50; 150.00]	0.046
Diastolic blood pressure (mmHg)	76.00 [76.00; 80.50]	90.00 [87.50; 90.00]	0.068
Delivery arms			0.400
Cesarean delivery	1 (33.33%)	3 (100.00%)	
Vaginal delivery	2 (66.67%)	0 (0.00%)	
Combination of antihypertensive drugs			0.100
No	3 (100.00%)	0 (0.00%)	
Yes	0 (0.00%)	3 (100.00%)	
Neonatal birth weight (g)	2930.00 [2,915.00; 3,260.00]	2020.00 [1865.00; 2675.00]	0.275
Sex of the newborn			1.000
Female	1 (33.33%)	2 (66.67%)	
Male	2 (66.67%)	1 (33.33%)	
X1 min Apgar	10.00 [10.00; 10.00]	9.00 [9.00; 9.50]	0.114
X5 min Apgar	10.00 [10.00; 10.00]	9.00 [9.00; 9.50]	0.114

### Overall grasp of pre-eclampsia-induced pathological alterations of the placenta

**FIGURE 1:**
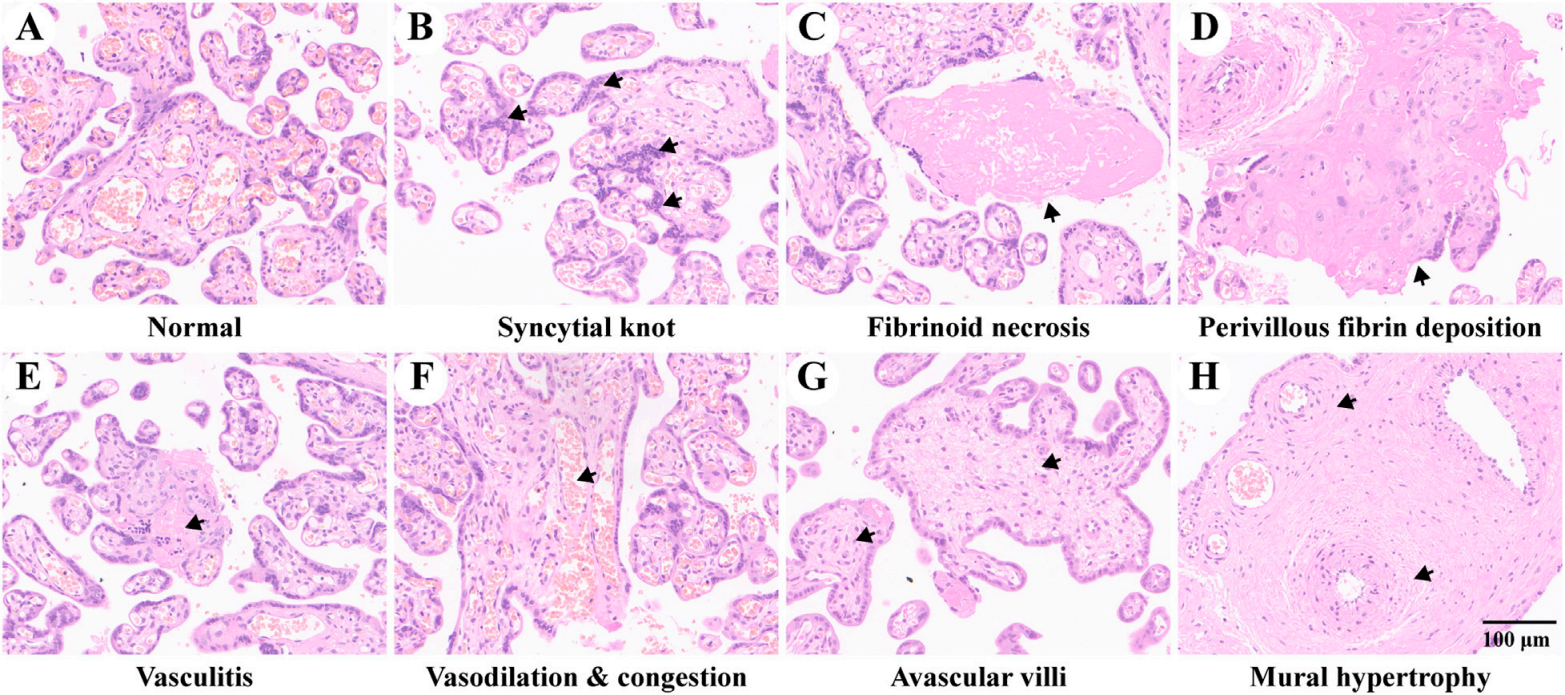
Placenta pathology of study subjects. **(A)** Placenta of normal pregnancy. B–H show placental pathological changes of pre-eclampsia. **(B)** Increased syncytial knots composed of aggregated syncytiotrophoblast between different villi. **(C)** Fibrinoid necrosis with hyalinization in the decidua. **(D)** Increased perivillous fibrin deposition. **(E)** Intravillous lymphohistiocytic infiltration of vasculitis. **(F)** Vascular vasodilation and congestion. **(G)** Avascular villi due to ischemia. **(H)** Mural hypertrophy as specific features of decidual arteriopathy. The representative pathological changes are indicated with black arrows.

Figure 1 shows an overview of the pathological characteristics of normal and pre-eclampsia placenta tissues, where the mature villi of the normal placenta are shown in Figure 1A, which were regular in shape with aggregated red blood cells in the vascular lumen, which was composed of trophoblasts. No placenta tissue was found to be with maternal and fetal vascular malperfusion, inflammatory cell infiltration, and abnormal pathological changes. In contrast, various specific pathological changes were identified in the placenta of pre-eclampsia patients and are shown in Figures 1B–H, including syncytial knot, fibrinoid necrosis, perivillous fibrin deposition, vasculitis, vasodilation and congestion, avascular villi, and mural hypertrophy, which might adversely affect the remodeling of spiral arteries and flow of blood, causing damage to maternal and fetal exchange of oxygen and nutrients.

### Identification of differentially expressed genes in pre-eclampsia

**FIGURE 2:**
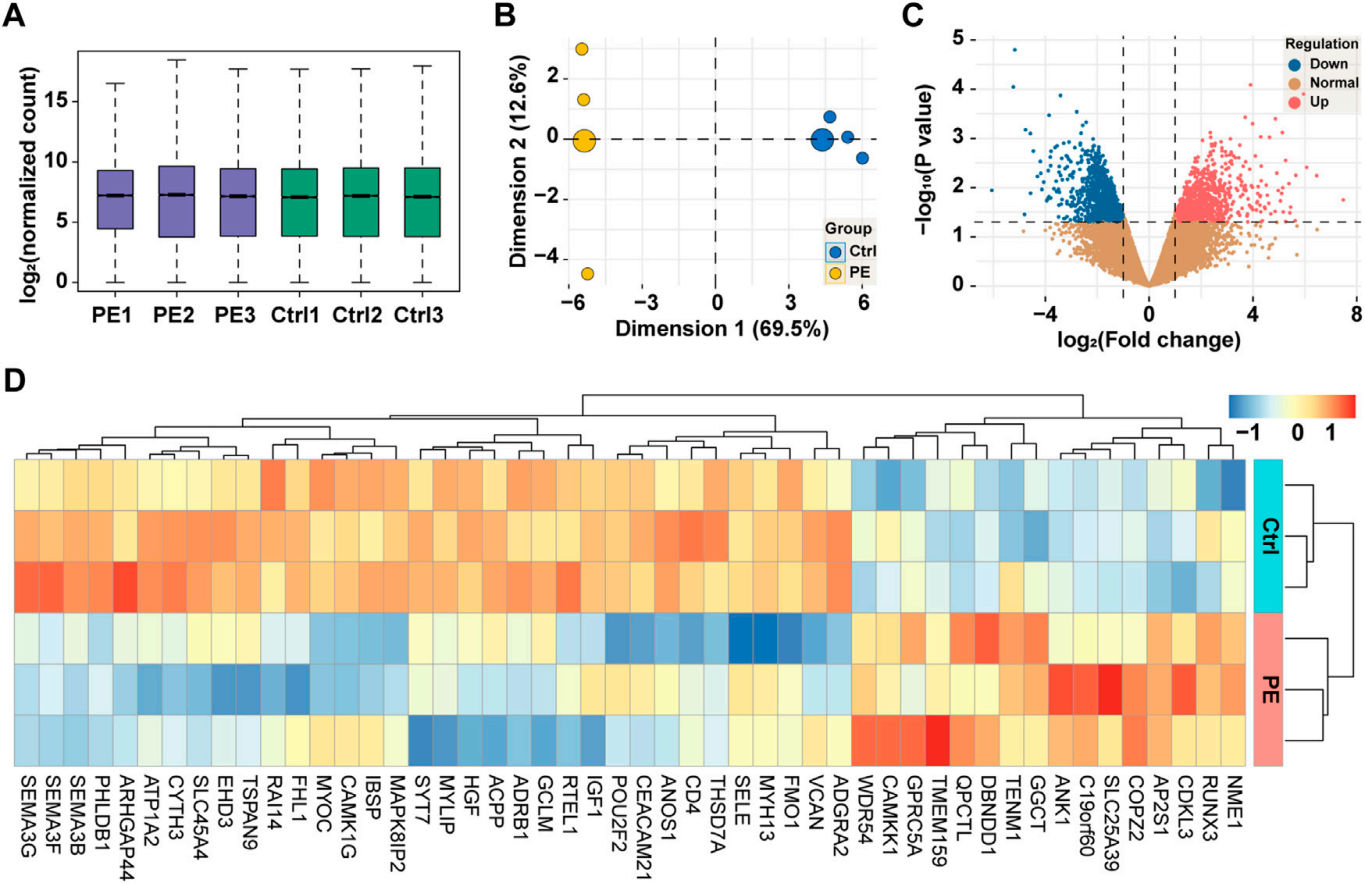
High-throughput sequencing of placental tissues. **(A)** Box-and-whisker plot showing the expression of genes in each sample. **(B)** Principal component analysis of samples based on the expression of genes at the genome-wide level. **(C)** Volcano plot showing the expression of genes between groups. Red, upregulation; blue, downregulation; yellow, normal. **(D)** Heatmap showing the expression of top 50 dysregulated genes with clustering analysis. PE, pre-eclampsia; Ctrl, control.

The expression of the total genes in each sample was visualized using a box-and-whisker plot as shown in Figure 2A, where all samples were uniformly distributed, and no outlier was observed. The result of the principal component analysis in Figure 2B showed that two principal components were determined in line with the grouping method. For comparison of the expression of genes between groups, a total of 22,133 genes were identified and are shown in Figures 2C–D, of which 960 genes were downregulated and 931 genes were upregulated. In addition, the top 50 dysregulated genes were represented by a heatmap, on which we exhibited the result of clustering analysis, and samples of the same group and genes in the same alteration trend were clustered together.

### Functional enrichment analysis of differentially expressed genes in pre-eclampsia

**FIGURE 3:**
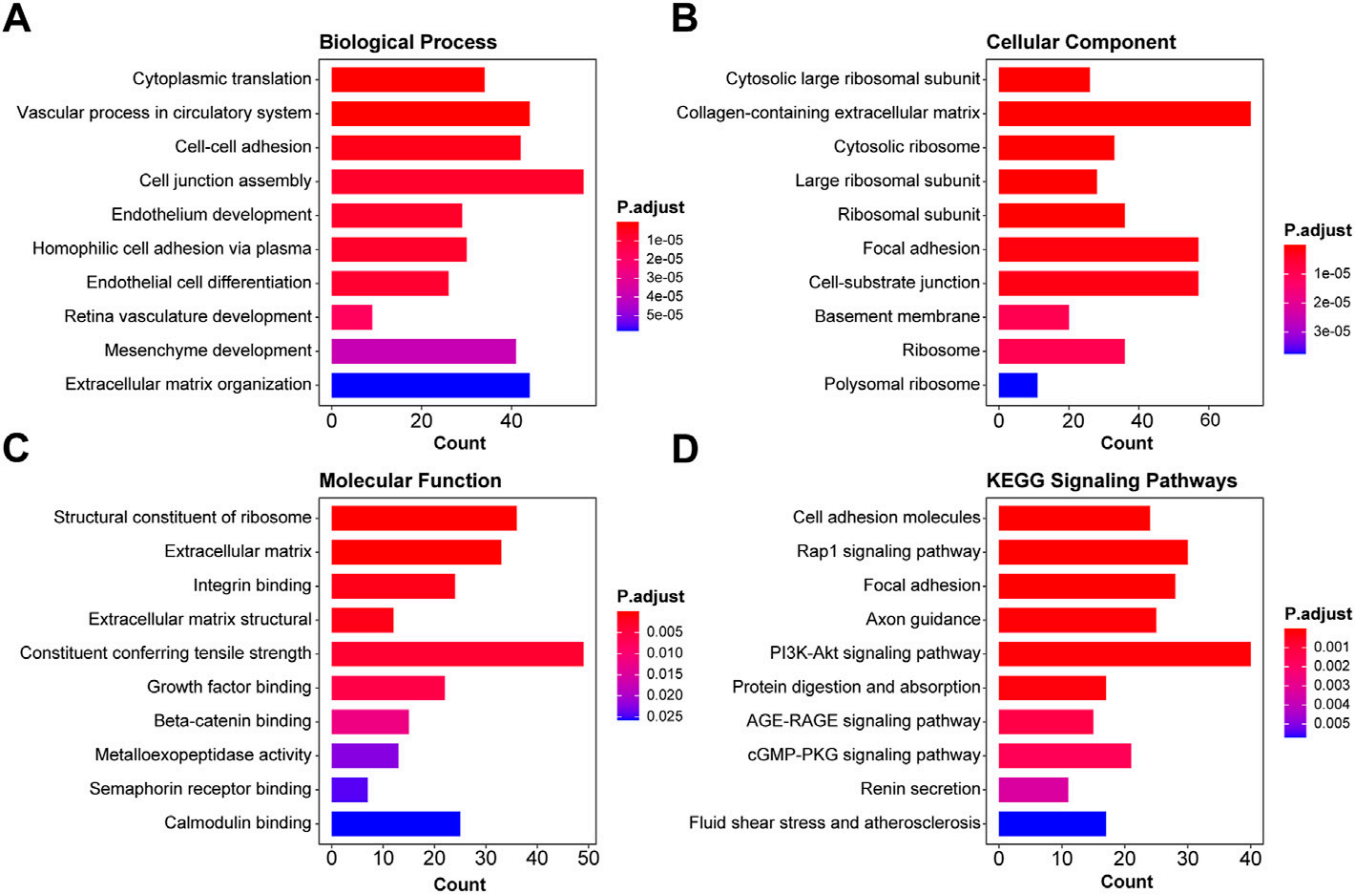
Functional enrichment analysis of differentially expressed genes in pre-eclampsia. Based on the 1,891 dysregulated genes, the functional enrichment analysis was conducted, and the top 10 GO enrichment items and KEGG items of interest were shown in the pattern of biological function **(A)**, cellular component **(B)**, molecular function **(C)**, and KEGG signaling pathways **(D)**.

Based on the differentially expressed genes, the functional enrichment analysis was conducted from two aspects, namely, GO and KEGG. For GO analysis, we examined the alterations of biological processes, molecular functions, and cellular components. As shown in Figures 3A–C, the top 10 items of GO analysis indicated that the dysregulation of 1,891 genes led to disorders in cytoplasmic translation, vascular process in the circulatory system, cell–cell adhesion via plasma membrane adhesion molecules, cell junction assembly, endothelium development, homophilic cell adhesion via plasma membrane adhesion molecules, endothelial cell differentiation, retina vasculature development in the camera-type eye, mesenchyme development, and extracellular matrix organization. These genes were mainly found in cytosolic large ribosomal subunit, collagen-containing extracellular matrix, cytosolic ribosome, large ribosomal subunit, ribosomal subunit, focal adhesion, cell–substrate junction, basement membrane, ribosome, and polysomal ribosome. The functional changes were structural constituent of ribosome, extracellular matrix structural constituent, integrin binding, extracellular matrix structural constituent conferring tensile strength, actin binding, growth factor binding, beta-catenin binding, metalloexopeptidase activity, semaphorin receptor binding, and calmodulin binding. KEGG enrichment analysis showed the related cell signaling pathways in Figure 3D, of which those signals of interest were cell adhesion molecules, Rap1 signaling pathway, focal adhesion, axon guidance, PI3K-Akt signaling pathway, protein digestion and absorption, EGFR tyrosine kinase inhibitor resistance, AGE-RAGE signaling pathway in diabetic complications, cGMP-PKG signaling pathway, renin secretion, and fluid shear stress and atherosclerosis.

### Disease enrichment analysis of differentially expressed genes in pre-eclampsia

**FIGURE 4:**
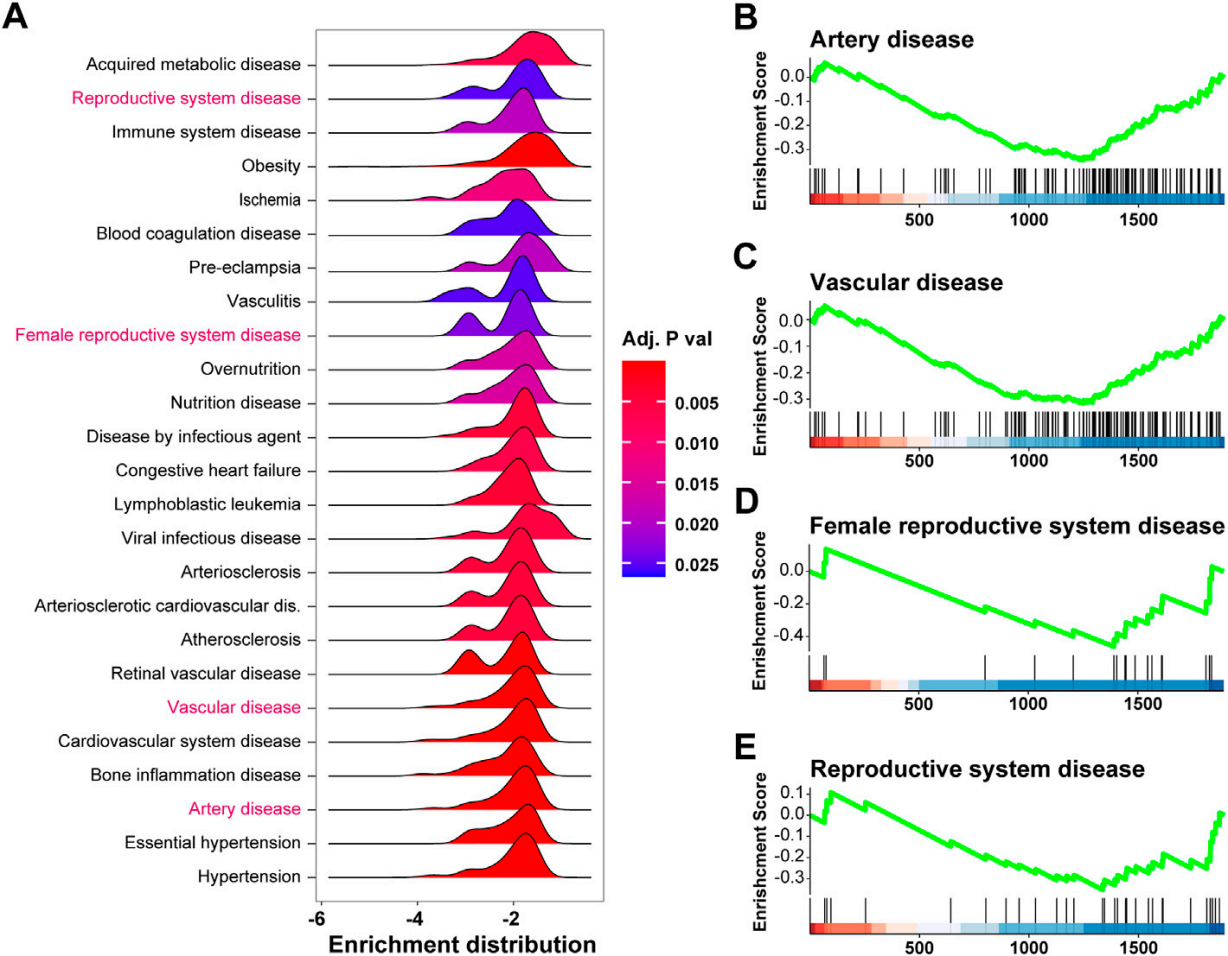
Disease enrichment analysis of differentially expressed genes in pre-eclampsia. The disease enrichment analysis and gene set enrichment analysis (GSEA) were conducted based on the expression profile of the 1,891 differentially expressed genes. **(A)** Ridge plot showing items related to immune response, reproduction, and vascular diseases in disease enrichment analysis. **(B–E)** GSEA plot showing the enrichment result of pre-eclampsia **(B)**, vasculitis **(C)**, vascular disease **(D)**, and female reproductive system disease **(E)**.

To investigate diseases potentially caused by the differentially expressed genes, we adopted disease enrichment analysis, by which the diseases were identified by semantic similarities among GO and GSEA items. As shown in Figure 4A, a total of 340 kinds of diseases were predicted to be associated with dysregulated genes, and items related to immune response, reproduction, and vascular diseases are shown in Figure 4A. Moreover, the results of GSEA on pre-eclampsia, vasculitis, vascular disease, and female reproductive system disease showed significant downregulation of related genes (Figures 4B–E).

### Downregulation of TEK/IGF1/CSF1/ANGPT2 promotes pre-eclampsia

**FIGURE 5:**
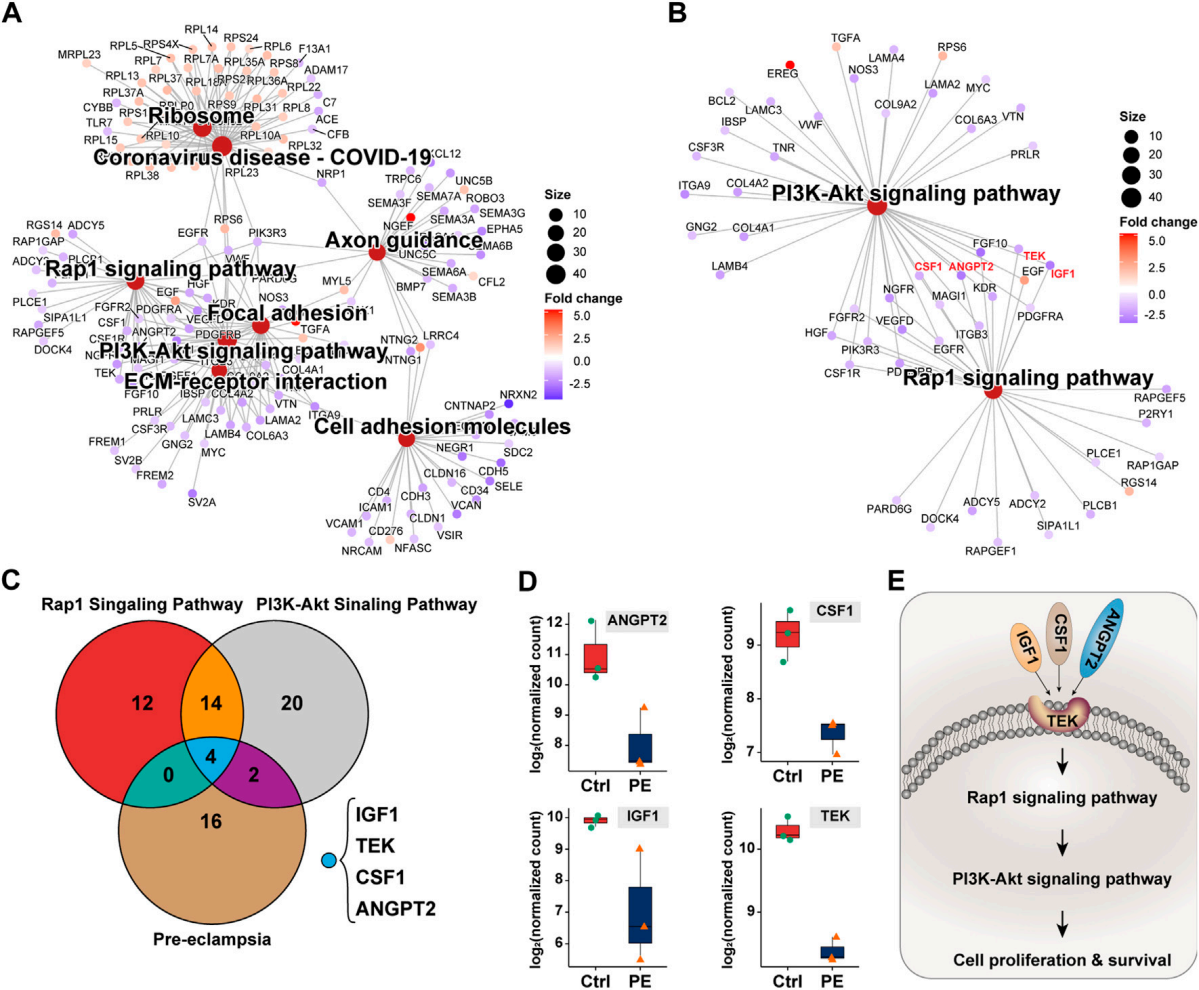
Deduction of the pathogenetic hypothesis of pre-eclampsia. **(A)** Topological network of KEGG signaling pathways of interest. **(B)** Topological network of the PI3K-Akt signaling pathway and Rap1 signaling pathway showing the overlapped differentially expressed genes. **(C)** Venn diagram showing the distribution of the differentially expressed genes between PI3K-Akt signaling pathway, Rap1 signaling pathway, and pre-eclampsia. **(D)** Box plot showing the expression and comparison of IGF1, ANGPT2, TEK, and CSF1 between groups of the control and pre-eclampsia. **(E)** Schematic illustration of IGF1/ANGPT2/TEK/CSF1-mediated pre-eclampsia.

**FIGURE 6:**
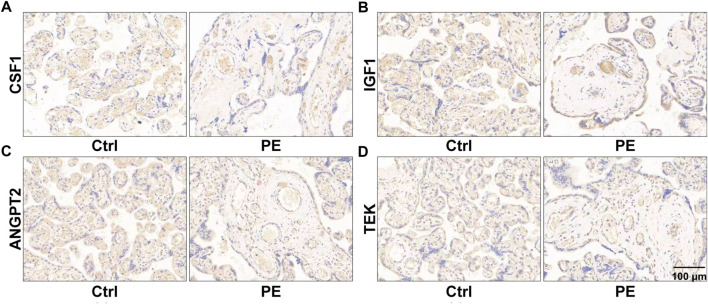
Images of immunohistochemistry showing the expression of placental CSF1 **(A)**, IGF1 **(B)**, ANGPT2 **(C)**, and TEK **(D)** in women with normal pregnancy and pre-eclampsia. The representative fields of the normal placenta for the control and lesion placenta for pre-eclampsia are shown.

To identify genes dominating the development of pre-eclampsia, we enrolled dysregulated genes of pre-eclampsia of disease enrichment analysis and signaling pathways of PI3K-Akt and Rap1, which were previously reported to mediate pre-eclampsia (Tang et al., 2020; Han et al., 2021). As shown in Figures 5A, B, a total of 18 genes were overlapped by the PI3K-Akt signaling pathway and Rap1 signaling pathway, namely, IGF1, HGF, NGFR, FGFR2, FGF10, ANGPT2, PDGFRB, PIK3R3, TEK, KDR, PDGFRA, EGF, EGFR, MAGI1, VEGFD, CSF1R, CSF1, and ITGB3. These genes were further intersected with pre-eclampsia, and four genes, namely, IGF1, ANGPT2, TEK, and CSF1 were identified and are shown in Figure 5C. Compared with the control group, the expression of these genes was downregulated at both transcriptional (Figure 5D) and prost-transcriptional levels (Figures 6A–D) in the placenta of pre-eclampsia patients. Given these data in collaboration with cellular component and KEGG enrichment analysis, which determined the biodistribution of the genes and associated signaling pathways, we visualized the pathogenetic hypothesis of pre-eclampsia as shown in Figure 5E.

## Discussion

Over a long period of history, pre-eclampsia has always been a common disease seriously threatening maternal and fetal lives. Despite great efforts made in conquering pre-eclampsia, it is still a tough work for early warning of pre-eclampsia before 20 weeks of gestation. Previous studies have shed light on the pathogenesis, diagnosis, and clinical treatment of pre-eclampsia, and several pathogenetic hypotheses regarding the development of pre-eclampsia are also raised, including immune disorder, placental maladaptation, and oxidative stress, but none of them can fully explain the etiology and pathogenesis of pre-eclampsia. This study is conducted with the aim to investigate all changes in the genes and identify potential targets dominating the development of pre-eclampsia. Successfully, we find that TEK, a membrane-embedded protein, is downregulated, thereby leading to the inhibition of trophoblast from sensing extracellular CSF1, IGF1, and ANGPT2 and causing suppression of cell proliferation and remodeling of spiral arteries.

In total, four genes, namely, TEK, CSF1, IGF1, and ANGPT2 are identified to be crucial for the pathogenesis of pre-eclampsia in this study. CSF1, also named M-CSF, is a colony-stimulating factor secreted by macrophages and plays an immunomodulatory role at the fetal–maternal interface. In a study conducted by Lindau et al. (2018), CSF1 is used as a growth factor for M2-like polarization, and, by skewing polarization of macrophages into a regulatory phenotype, CSF1 is found to promote the establishment of the tolerant milieu at the fetal–maternal interface. However, in a nested case–control study, Nick and colleagues tested the serum levels of M-CSF, VEGF, and placental growth factor (PlGF) of 23 pregnant women partially with pre-eclampsia, and they report that the alteration of M-CSF is of no statistical significance between groups (Bersinger and Ødegård, 2005).

IGF1 is functionally and structurally similar to insulin at the protein level and participates in mediating growth and development. In an epidemiological study based on pregnant women, Kanninen et al. (2014) detect the serum levels of IL-13, TGF-β1, and IGF1 and find lower levels of IGF1 (14.9 ng/ml) in pre-eclampsia than the controls (18.4 ng/ml), but decreased IGF1 has no association with autophagy. In addition, the serum IGF1 at 11–13 weeks of gestation is also reported to be of no predictive effects on small gestational age neonates (Sifakis et al., 2012). Sharon shows more direct evidence on IGF1 in predicting pre-eclampsia based on 1 650 low-risk Caucasian women, and they insist on no significant correlation between maternal IGF1 and gestational hypertension or pre-eclampsia (Cooley et al., 2010). In contrast, Haruki finds that the expression of cleave insulin-like growth factor-binding proteins (IGFBPs) is responsible for the suppression of the IGF signaling pathway, and the suppression of IGF can be compensated by the upregulated PAPP-A2 (Nishizawa et al., 2008). Taking the studies mentioned previously into consideration, it is clear that the role of IGF1 in the development of pre-eclampsia is still under debate. Here, we find that the placental IGF1 is downregulated by 2.95 folds in pre-eclampsia as compared to the control, largely differs from the serum IGF1 levels, and this might also be the reason why serum IGF1 is of limited predictive effects for pre-eclampsia.

ANGPT2 belongs to the angiopoietin family of growth factors, and it is an antagonist of angiopoietin 1, a ligand for endothelial TEK, in its protein form. The association of ANGPT2 with pre-eclampsia has also been studied in previous studies, and ANGPT2, in collaboration with PlGF, sFlt-1, and sEng, is suggested to be essential for placental vessel development. Furthermore, an imbalance of ANGPT2 causes dysregulation of placental vessel remodeling, and the inhibition of ANGPT2 can lead to an anti-angiogenic state in the placenta and contribute to the development of pregnancy pathologies (Umapathy et al., 2020). Based on the serum expression levels of ANGPT2, AFP, PlGF, RBP4, and sTNFR1 at the first trimester, Jaana establishes a predictive model for early onset pre-eclampsia, but ANGPT2 was not retained in the final model (Nevalainen et al., 2017). Similarly, by observing the longitudinal changes of maternal plasma levels of sEng and ANGPT2, Khalil et al. (2014) report that the square root of ANGPT2 decreases significantly with gestational age, but there is no difference between groups of normotensive, hypertension, pre-eclampsia, and preterm pre-eclampsia. Hence, the downregulation of ANGPT2 identified in this study may be a result of shortened gestational weeks of the pre-eclampsia group, which should be treated seriously when being used as a screening biomarker for pre-eclampsia.

By binding to CSF1, IGF1, and ANGPT2, TEK plays a crucial role in the development of pre-eclampsia, and it also encodes a receptor that belongs to the protein tyrosine kinase Tie2 family. Often, TEK is studied accompanied by ANGPT2. For example, in a prospective study aiming to identify specifics of pre-eclampsia, the serum VEGF, ANGPT2, and TEK were tested and compared, where TEK was found to be downregulated in the serum of pregnant women with pre-eclampsia and small growth for gestational age (Aref et al., 2013).

To sum up, utilizing the placenta donated by six pregnant women (three control and three pre-eclampsia), we profiled the expression of genes at the genome-wide level and found 1,891 dysregulated genes, based on which we proceeded with the bioinformatics analysis and raised the pathogenetic hypothesis of pre-eclampsia. By focusing on the PI3K-Akt signaling pathway, Rap1 signaling pathway, and disease enrichment analysis item pre-eclampsia, TEK, CSF1, IGF1, and ANGPT2 were identified to promote the development of pre-eclampsia. After confirming the placental expression of these genes at the protein level, we proposed the pathogenesis of pre-eclampsia that the downregulation of TEK, CSF1, IGF1, and ANGPT2 may inhibit trophoblast proliferation and affect the remodeling of spiral arteries, causing maternal and fetal malperfusion and impeding nutrient exchange, thereby leading to clinical manifestations of pre-eclampsia.

## Data Availability

The datasets for this article are not publicly available due to concerns regarding participant/patient anonymity. Requests to access the datasets should be directed to the corresponding author.
